# The Case for an Expanded Concept of Trained Immunity

**DOI:** 10.1128/mBio.00570-18

**Published:** 2018-05-22

**Authors:** Antonio Cassone

**Affiliations:** a Polo della Genetica, Genomica e Biologia, University of Perugia, Perugia, Italy; GSK Vaccines

**Keywords:** *Candida*, immunity, inflammation, innate

## Abstract

Trained immunity was originally proposed as a program of innate immunity memory by innate immunity cells of hematopoietic origin such as the monocytes/macrophages and the NK cells. Here I discuss some old and new data justifying this program and some specific, still unanswered, questions it raises regarding the model fungus Candida albicans and the chronic, inflammatory vulvovaginal disease it causes. Building upon this well-established program, the recent reports that epithelial cells of mammals can also acquire memory from previous stimulations, and the apparent intrinsic ability of many living cells from bacteria to mammals to learn from experience, I suggest an expansion of the concept of trained immunity to include all cells of different lineages with the potential of memorizing previous microbial encounters. This expansion would better fit the complexity of innate immunity and the role it plays in infectious and inflammatory diseases.

## PERSPECTIVE

### Trained immunity.

Trained immunity (TI) is a persistent (weeks to months) immunomodulation of cells of innate immunity of hematopoietic lineage, particularly monocytes/macrophages, due to memory acquisition upon microbial or nonmicrobial stimulation. In the field of infectious diseases, the outcome of this immunomodulation is a modified (usually enhanced) secondary response to an already encountered pathogen coupled with nonspecific responses to unrelated pathogens and even nonmicrobial antigens.

While the existence of an “adaptive” or “memory” component of innate immunity was suspected or suggested (1, 2), Netea and collaborators first proposed the term trained immunity to conceptualize all evidence for an anti-infectious, protective, memory response entirely mediated by cells of innate immunity (3–5). TI is a notable changing concept, since memory responses had long been considered to be an exclusive property of adaptive immunity. TI could be seen as a bridge between the classically defined innate (no or low specificity, no memory) and adaptive (high specificity, memory) immunity. Actually, it adds another conceptual piece of evidence for the high flexibility and plasticity of innate immunity (1).

It is recognized that training the cells of innate immunity to make them acquire memory and other adaptive functions is an evolutionarily conserved biological property, from some primitive forms of it in invertebrates to well-developed ones in mammals (6–8). Common to all organisms also appears to be the molecular basis of the phenomenon which relies upon specific epigenetic reprogramming of innate immune cells, with chromatin remodeling, changes in gene transcription, and a profound metabolic shift (9, 10). The short half-life of circulating and tissue-resident macrophages contrasts the persistence of their trained memory and suggests that the epigenetic reprogramming occurs in the bone marrow progenitors which give rise to lines of terminal memory cells (3, 11). TI is currently advocated to underlie most of the heterologous effects of vaccination, i.e., its capacity of stimulating immune responses to nontarget antigens, particularly those observed with attenuated vaccines such as *Mycobacterium bovis* BCG, measles, and oral polio (12, 13). Recently, microglial priming has also been suggested to be an example of trained immunity (14).

### TI in retrospect.

In mammals, the definition of TI and mechanistic insights are rather recent (3, 9, 10). However, a few basic observations in experimental murine models, now widely recognized as suggestive of, if not frankly attributable to TI, date back to research performed in the early 1980s (15–17). A short recapitulation of those findings can help highlight aspects of TI, particularly its relationship with immune priming and immune tolerance, that are still unclear.

In our initial search for a *Candida* vaccine, we addressed mouse vaccination with a low-virulence, nongerminative strain of Candida albicans causing a mild, chronic infection (strain PCA2 [18]) and showed that the mice were protected from a lethal challenge with a fully virulent C. albicans strain, as usually happens with other protective, attenuated bacterial and viral vaccines. Surprisingly (at that time), the PCA2-immunized animals were also protected from other virulent *Candida* species, other fungi such as *Aspergillus* and *Cryptococcus*, and even bacteria (Staphylococcus aureus). Adoptive transfer experiments demonstrated that PCA2-activated macrophages were responsible for protection (15).

The experimental design of the above studies does not allow for a sharp distinction between persistent immune priming and TI. However, the protective macrophages were not armed by *Candida*-specific T cells, as high protection was also observed in animals given immunosuppressive drugs as well as in athymic, nude mice (16, 17). Furthermore, the long capacity of splenic macrophages of vaccinated mice to maintain an interleukin 1 (IL-1)-dominated cytokine profile and microbicidal activity *in vitro* (17) clearly tips data interpretation toward a real TI. In fact, a macrophage durable activation and inflammasome-dependent IL-1β production (see also below) have been amply confirmed and extended as paradigmatic attributes of C. albicans-induced TI (19, 20).

An aspect of our investigations that also received little attention comes from the observation that the PCA2-immunized mice, while rapidly eliminating the virulent strain of C. albicans (and the cohort of other microbial challengers), remained chronically infected by the immunizing agent (15–17). The question is why a robust and wide-spectrum macrophage activity unchained by TI was unable to eliminate a low-virulence fungus. Is the commensal nature of C. albicans, a microorganism suited to exploit (or induce?) host tolerant tissues the sole reason or is the phenomenon common to immunization with other attenuated vaccines, causing low-grade, chronic infections when the host is challenged by virulent pathogens? While these questions remain substantially unanswered, it is notable that BCG, which also causes mild chronic infection for up to 1 month after vaccination, brings about high protection from various other agents unrelated to the vaccine target (12, 13).

Issues of interest are also the interconnections between TI, immune tolerance, and inflammatory diseases. Some of the epigenetic reprogramming of cells of innate immunity that occurs during immune tolerance induced by lipopolysaccharide (LPS) seems to recognize the same basic mechanisms as those characterizing TI, and importantly, excess training can lead either to tolerance or inflammatory diseases (21). It is possible that a degree of overlap also exists between immune priming and TI, as shown by the interesting phenomenon of transgenerational transmission of anti-infectious resistance in the red flour beetle Tribolium castaneum infected by Bacillus thuringiensis (22). Overall, the real, mechanistic differences between TI and persistent immune priming are still to be investigated. As usual in biology, the borders defining complex phenomena remain tenuous. There is a need to address the mechanisms that make these borders permeable, as this can improve our understanding of the role of innate immunity in antimicrobial defense and vaccination. For these studies, a model fungus like C. albicans, which can occupy several host niches as an opportunistic pathogen or commensal, and can express several inducers of immune priming, inflammation, and TI (beta-glucan, chitin, and some virulence proteins [23–26]) could provide clear advantages.

### About memory and immunity.

As discussed above, immunologic memory can be acquired by cells of innate immunity of hematopoietic origin following a suitable training, and this has important biological consequences. However, the words immunity and memory have been used in a more extensive way in areas that have apparently nothing to do with immunology. Just one example is the bacterial immunity to virus attacks and the ability of the survivors to remind virus-specific sequences, such as the clustered regularly interspaced short palindromic repeat (CRISPR) (27). Behaviors implying some form of memory can easily be found in amoebic organisms, low fungi and plants, with a realistic suspicion that they could be much more common and pervasive than previously thought (5–8). A perhaps naive view of all these phenomena could suggest that, in principle, every living cell might be capable of learning from experience, storing for a time by its own epigenetic reprogramming what has been learnt, and display and use it when needed. More conservatively, could we extend the immune priming and TI concepts to at least all cells of innate immunity, including those which are not of hematopoietic origin?

### Epithelial cells and inflammasome.

The cellular armamentarium of innate immunity is not exhausted by phagocytes and NK cells. In particular, the epithelial cells (EC) play a fundamental role in protecting against infection, acting both as physical barrier and as producers of antimicrobial compounds (28, 29). EC are also suitable niches for colonization by safe and/or opportunistic pathogens, capable of instructing an interactive talk with the host. EC respond to colonization and infection using receptors for microbial sensing and discrimination between pathogens and commensals and then signaling for a response that, depending on the kind of pathogen/colonizer and the tissue involved, can maintain homeostasis, help clear the offender, or be pathogenic. Some biochemical insights into the discriminative EC response to a pathogen/commensal encounter such as C. albicans have been reported (30, 31). The final response to the pathogen is tissue inflammation, the outcome of which can be quite divergent in different tissues, as exemplified by the marked distinction of the role of EC and inflammation in oral versus vaginal C. albicans colonization and disease (28). Unfortunately, little attention has been devoted to understand the specific tissue inflammatory reactions to various pathogens or commensals. Nonetheless, the core of the inflammatory response rests on the induction and activation of one or more intracellular EC receptor complexes called inflammasomes, with their downstream cytokine effectors of the IL-1 family. Gene expression of NLRP3 inflammasome components, in particular caspase 1, by the vaginal EC has been recently reported to be the distinctive hallmark of human vulvovaginal candidiasis, substantiating previous, suggestive genetic or experimental evidence (32–36). In this context, it would be of great interest to assess the potential role of another “epithelial” inflammasome, the NLRP6, which in intestinal EC, regulates the microbiota homeostasis and inflammation (37). Remarkably, inflammasomes also play a central role in the memory response of monocytes/macrophages (9).

In principle, all of the above suggest that EC can do more in their relationship with pathogens than previously thought. In particular, do EC also exploit TI-like mechanisms to modulate their responses to successive encounters with a pathogen? Recent reports suggest they do indeed, by means of epigenetic mechanisms similar to those underpinning monocyte/macrophage training. Nalk and collaborators (38) have shown, in a mouse model of imiquimod-induced inflammation and wound healing, that faster healing occurs in those animals that were previously exposed to the inflammatory stimulant, an effect recalled by other, unrelated stimulators, including C. albicans, and mediated by long-lived epithelial stem cells. Experiments of depletion of skin-resident macrophages and ablation of T and B cell responses demonstrated that the epithelial stem cells, not any other cell type resident or infiltrating the skin, were indeed responsible for the accelerated healing (38). The data suggest that mature EC can inherit the memory of inflammation by the epithelial stem cells residing in the basal layer of the columnar epithelia. A different but somewhat related example has been reported by Nasrollahi and collaborators (39) who showed that EC migration during tissue regeneration can be primed to accommodate for different matrix stiffness. Noteworthy, EC can memorize previous knowledge of a given matrix stiffness, a property that has been called “mechanical memory” (39).

### An expanded concept of trained immunity for antimicrobial defense.

The observation that mucosal EC can be primed for memory responses adds to the training of other cells of innate immunity in opening important perspectives for the control and transmission of infectious disease and vaccination. EC are the prime external or internal body contact with the microbial world, and the possibility that these cells or part of them can memorize the above contacts raises particularly relevant and novel biological issues. EC memory can cooperate or interfere with memories of innate and adaptive immunity in the outcome and persistence of protective immune responses and in the microbiota homeostasis. Candida and candidiasis are a special case in point, since this fungus is a normal commensal of the intestinal and vaginal mucosa, while as an opportunistic agent, it can cause high-prevalence infections of oral and vaginal mucosa. Besides its potential role in favoring new approaches to a much-needed anti-*Candida* vaccine (40), EC memory could play a role in the commensalism of the normal host by this fungus, some intrinsic capacity EC have to keep it at bay (24) and in the onset and recurrence of disease. Modulations of EC memory could be particularly involved in recurrences of vulvovaginal candidiasis (VVC), a disease characterized by bouts of unresolvable pathogenic inflammation associated with a substantial anergy of the vaginal recruited neutrophils, which usually are the most potent candidacidal effectors (41). Notably, all these events occur in a setting of normal functioning of adaptive immunity. The overall picture of recurrent VVC almost perfectly fits the recognized pattern of other inflammatory diseases and immune tolerance where the roles of EC, inflammasomes, neutrophils, and commensal microorganisms have long been underscored (42). An outstanding addition now appears to be the EC inflammatory memory.

### A conclusive hypothesis.

Building on the already well-developed TI concept and the recent evidence for EC stem cell memory and speculating about the apparent intrinsic attitude of living cells to acquire some memory from experience, I am here proposing an expansion of the TI concept (expanded trained immunity [ETI]) that considers all cells involved in antimicrobial defense, both of hematopoietic and nonhematopoietic origin, as a unique, interactive, cross talking cellular “organism” sharing memories of previous microbial encounters. ETI is an exciting perspective for novel thinking and improved approaches to the fight against infectious and inflammatory diseases.
